# An overview of glucagon-like peptide-1 receptor agonists for the treatment of metabolic syndrome: A drug repositioning

**DOI:** 10.22038/ijbms.2020.41638.9832

**Published:** 2020-05

**Authors:** Maryam Rameshrad, Bibi Marjan Razavi, Jean-Daniel Lalau, Marc E. De Broe, Hossein Hosseinzadeh

**Affiliations:** 1 Natural Products and Medicinal Plants Research Center, North Khorasan University of Medical Sciences, Bojnurd, Iran; 2 Targeted Drug Delivery Research Center, Pharmaceutical Technology Institute, Mashhad University of Medical Sciences, Mashhad, Iran; 3 Department of Pharmacodynamics and Toxicology, School of Pharmacy, Mashhad University of Medical Sciences, Mashhad, Iran; 4 Université de Picardie Jules Verne, Department of Endocrinology, Amiens, France; 5 Universiteit Antwerpen, Department of Biomedical Sciences, Antwerpen, Belgium; 6 Pharmaceutical Research Center, Pharmaceutical Technology Institute, Mashhad University of Medical Sciences, Mashhad, Iran

**Keywords:** Diabetes, Dyslipidemia, GLP-1 receptor agonist, Hypertension, Metabolic syndrome, Obesity

## Abstract

Metabolic syndrome (MetS) is a clustering of several cardiovascular risk factors that include: obesity, dyslipidemia, hypertension and high blood glucose, and often requires multidrug pharmacological interventions. The management of MetS therefore requires high healthcare cost, and can result in poor drug treatment compliance. Hence drug therapies that have pleiotropic beneficial effects may be of value. Glucagon-like peptide-1 receptor agonists (GLP-1RAs) are the newest anti-diabetic drugs that mimic incretin effects in the body. They appear to be safe and well tolerable. Herein, the pharmacology of GLP-1RAs, their side effects, drug interactions and their effects in MetS is assessed. We conducted a Google Scholar, PubMed, Scopus, and Web of Science search since 2010 to identify publications related to the use of GLP-1RAs in treating component features of the MetS. Keywords used for the search were: GLP-1 receptor agonist, exenatide, liraglutide, lixisenatide, albiglutide, dulaglutide, MetS, obesity, triglyceride, cholesterol, lipid, hypercholesterolemia hyperlipidemia, atherosclerosis, hypertension, blood pressure, hyperglycemia, hypoglycemia and blood glucose. According to the gathered data, GLP-1RAs appear safe and well tolerated. Pre-clinical and clinical studies have evaluated the lipid-lowering, anti-atherosclerotic, anti-hypertensive and anti-diabetic effects of this class of drugs. Some these effects are related to a reduction in food-seeking behavior, an increase in atrial natriuretic peptide level and hence vascular relaxation and natriuresis, and an increase of pancreas β-cell mass and protection against glucotoxicity. Collectively, this review indicates that there may be some value in GLP-1RAs repositioning to manage MetS risk factors beyond their anti-diabetic effects.

## Introduction

Incretin peptides, gastric inhibitory polypeptide (GIP) and glucagon-like peptide (GLP)-1, are secreted by the enteroendocrine cell populations known as K-cells and L cells, respectively. They stimulate pancreatic insulin secretion in response to food ingestion and enhance glucose-dependent stimulation of insulin secretion that is known as the “incretin effect” (1, 2). GIP and GLP exert their insulinotropic effects via G-protein-coupled receptors that are mainly expressed on pancreatic β cells. These receptors are also expressed in peripheral tissues and are responsible for extra pancreatic actions of incretin hormones and their metabolic effects (3). The incretin effect is responsible for 50-70% of total insulin secretion after glucose ingestion (1, 2) and is often reduced in patients with type 2 diabetes mellitus (T2DM) (4). GLP but not GIP secretion is also reduced in T2DM (4). Glucagon-like peptide-1 receptor agonists (GLP-1RAs) with extended biological half-life have been introduced as a new class of antidiabetic drugs.

The metabolic syndrome (MetS) is a clustering of metabolic disorders. It may be defined by the presence of three out of five from the following medical conditions: elevated fasting plasma glucose, abdominal obesity, elevated blood pressure, high serum triglycerides and low high-density lipoprotein (HDL) levels (5). 

A growing body of evidence supports the idea that MetS is due to the combination of genetic factors (6, 7) and lifestyle factors such as diet (8) and sedentary behaviors (9). Furthermore, exposure to some chemicals (10) could increase the incidence of MetS in special occupations (11). Previous study has shown significant association of MetS with anxiety and depression (12). Excess energy intake and obesity have a pivotal role in the development of MetS components including elevated blood pressure, insulin resistance and hyperglycemia, pro-thrombotic state, pro-inflammatory state and atherogenic dyslipidemia. In this regard, non-pharmacological approaches i.e., lifestyle modification, caloric restriction are considered the primary interventions for the treatment of the syndrome (13, 14). Herbs and dietary poly phenols (9) i.e., rutin (15), black seed (16), garlic (17), grapes (18), rosemary (19), cinnamon (20), saffron (21), green tea (22), berberine (23), pepper (24), mangosteen (22) and avocado (23) may partly influence it. Besides, modification of gut microbiome by the use of prebiotics, probiotics or other dietary interventions (25) is worthful. However, in the case of severe obesity, bariatric surgery and drug therapies may be necessary. Depending on the individual patients risk profile different pharmacological drugs are prescribed in MetS (14). Since metabolic syndrome is a multifactorial and complex disease, a combination therapy is needed to manage it. However, this kind of therapy reduces patients adherence and increases health cost and the chance of drug interactions (9). It is useful to have drugs with multiple effects, but they often are insufficiently potent to treat all the features of MetS. Recently a review has focused on DPP-4 inhibitors as a choice in managing some levels of MetS due to their polypharmacologic effects (26). However, there are limited studies on chemical drugs with pleiotropic effects for managing MetS. The aim of this study is filling this gap by introducing GLP-1RAs as a worth full drugs in metabolic syndrome that increases the number of individual targets and decreases cost and side effects of multiple therapies in this disease. 

## Methods

The relevant data were collected by searching the Google Scholar, PubMed, Scopus, and Web of Science. The keywords used as search terms were GLP-1 receptor agonist, exenatide, liraglutide, lixisenatide, albiglutide, dulaglutide, metabolic syndrome, obesity, triglyceride, cholesterol, lipid, hypercholesterolemia hyperlipidemia, atherosclerosis, hypertension, blood pressure, hyperglycemia, hypoglycemia and blood glucose. 

All kinds of preclinical (*in vitro*, *in vivo*) and clinical studies that have been published since 2010 were included. Furthermore, bibliographies of eligible articles were examined for additional relevant studies. Nevertheless, congress abstracts, as well as non-English language studies, were considered ineligible for inclusion. 

Based on the method, 129 appropriate articles were selected from about 750 articles that were gathered from the first search. Selected data were categorized in the following main headings “pharmacology of GLP-1 receptor agonists” and “the effects of GLP-1 receptor agonists on metabolic syndrome”.

***Pharmacology of GLP-1 receptor agonists***

*Pharmacodynamics and pharmacokinetics*

GLP-1 is a 30 or 31 amino acid long peptide that is processed from proglucagon (27). GLP-1 in pancreatic β-cells increases the mass and decreases apoptosis and drives insulin secretion in a glucose-dependent manner that decreases the risk of hypoglycemic episodes. It does not increase insulin secretion when the blood glucose is low. GLP-1 action on pancreatic α-cells inhibits glucagon secretion. Apart insulinotropic and glucagon suppressing effects, GLP-1 evokes a good control on postprandial glucose attributed to slowing gastrointestinal motility that in turn leads to delay in absorption of glucose into the circulation. Besides, by inducing central satiety, it reduces food intake and results in weight loss. (28). However, the half-life of active GLP-1 is very short, 2 min, due to enzymatic degradation especially by dipeptidyl peptidase (DPP)-4 (27). According to the aforementioned data, GLP-1RAs which are resistant to degradation by DPP-4 enzyme have been developed for managing T2DM. They mimic the effects of the incretin hormone GLP-1 with longer duration action and greater potency for glucose-lowering than it. Like insulin, these drugs are given by injection. Currently available GLP-1RAs in the market are derivatives of either human GLP-1 (liraglutide, albiglutide, and dulaglutide) or exendin-4 (exenatide, lixisenatide, and exenatide-long-acting release). Furthermore, GLP-1RAs differ with each other based on pharmacokinetic properties and pharmacodynamics profile (28, 29) (Table 1).

Long-acting GLP-1RAs with several days’ half-life are prescribed weekly. They consist of albiglutide, dulaglutide, exenatide-long-acting release (28) and recently approved ones, semaglutide (30). Short-acting forms have a plasma half-life of 2-12 h. They include exenatide, lixisenatide and liraglutide that are administrated daily (28). Of all GLP-1RA drugs that are prescribed subcutaneously, only semaglutide has the potential to be administered in an oral formulation. Studies on this oral agent are being evaluated (30). 

Long-acting GLP-1RAs predominantly influence on both insulin and glucagon secretion that in turn regulate pre- and post-prandial glucose level. Besides, they have strong effects on fasting blood glucose. But, the short-acting agents mostly affect on the gastric emptying rate and so post-prandial glucose levels (28, 29). 

The efficacy of the long-acting release formulation of exenatide in the improvement of glycemic control and hemoglobin A1c (HbA1c) reduction is greater than its twice-daily formulation (31). However, liraglutide once daily provides greater improvement in glycemic control than does once-weekly administration of exenatide (32) or albiglutide (33). Besides, comparing liraglutide once a day with exenatide twice a day showed a significantly greater improvement in glycemic control with liraglutide especially in obese diabetic patients (34). Administration of lixisenatide once daily is not superior to exenatide twice daily in HbA1c reduction (35). Besides, once-weekly dulaglutide has no greater reduction in HbA1c in comparison with once-daily liraglutide (36). Semaglutide efficacy in controlling glycamia and body weight is greater than the other GLP-1RAs of exenatide releases and dulaglutide (37)(Table 1).

Pharmacogenetic studies on GLP-1R agonists should be considered. The presence of naturally occurring nonsynonymous single nucleotide polymorphisms on GLP-1 gene, evokes multiple complexity and effect on the pharmacological impact of GLP-1RAs. Some introduced human GLP-1R single nucleotide polymorphisms are as follow: substitution of Leu for Pro at position 7 (rs10305420), substitution of Ley for Arg at position 20 (rs10305421), substitution of His for Arg at position 44 (rs2295006), substitution of Gln for Arg at position 131 (rs3765467), substitution of Met for Thr at position 149 (112198), substitution of Ser for Gly at position 168 (rs6923761), substitution of Leu for Phe at position 260 (rs1042044), substitution of Thr for Ala at position 316 (rs10305492), substitution of Cys for Ser at position 333 (rs10305493), and substitution of Gln for Arg at position 421 (rs10305510). Rs10305493 variant preserves the function of GLP-1R (38). However, Rs367543060 variant dramatically decreases the peptide response (38, 39). In non-diabetic volunteers, rs6923761 variant, the substitution of serine for glycine at position 168, decreases insulinotropic responses to GLP-1 infusion in hyperglycemic condition (40). In contrast, this polymorphism increases the efficacy of liraglutide on weight loss and metabolic improvement in diabetic patients (41).

*Side effects*

Comparing with long-acting exenatide, mild nausea, vomiting, upper respiratory tract infections and injection site bruising are more frequent with that twice a day. Injection site pruritus with a mild intensity that is resolved with continued treatment and constipation is seen with long-acting exenatide (31). Besides, the frequencies of gastrointestinal disturbances (nausea, diarrhea, and vomiting) with the long-acting formulation of exenatide are greater than liraglutide (32). Both liraglutide (34) and lixisenatide (35) are well tolerable than exenatide twice daily formulation. Compare with albiglutide, the rate of nausea and vomiting is lower and injection-site reactions are greater than liraglutide (33). The tolerability of dulaglutide and liraglutide seems to be the same (36)(Table1).

Totally, all GLP-1RAs are well tolerable and incidence of adverse effects is low. Gastrointestinal problems and nausea are the most commonly reported complications with them that are minimized by gradual dose titration. In exenatide-treated patients, anti-exenatide antibody formation has been reported (42). Pancreatitis and pancreatic cancer, thyroid C cell tumors, gallbladder-related adverse events (43) and retinopathy (with semaglutide) (30, 44) are the other concern about GLP-1RAs that further studies should determine them.

*Drug interactions*

Since this class of drugs prolongs the gastric emptying time, the availability of gastric material to small intestine decreases. So, in combination therapy with other drugs, medication absorption slows, C_max_ decreases and t_max_ delays (45). Therefore, in combination therapy of exenatide with warfarin, warfarin dose adjustment is recommended (46). However, delaying gastric emptying time with GLP-1RAs may increase the chance of solubility and dissolution of some drugs in gastric juice that results in increased C_max_ (45). This effect is seen in combination therapy of liraglutide with griseofulvin (47). Totally, GLP-1RAs-drug interactions are important with drugs that require a rapid start of action or those need to reach a suitable concentration peak (45).

***The effects of GLP-1 receptor agonists on metabolic syndrome***

*Effects on lipid profile, body weight and related complications*

Atherogenic dyslipidemia is defined as the elevation of low-density lipoprotein (LDL-C) and non-HDL-C accompanied by the decrease of HDL-C. This condition results in atherosclerosis and cardiovascular diseases. Insulin resistance, obesity, and related inflammatory conditions are associated with atherogenic dyslipidemia and atherosclerosis (5, 48). Obesity and dyslipidemia are the main predisposing factors for MetS. Incretin-based therapy is now considered as a new approach to treating obesity (2, 49) concurrently with diabetes (50) or associated with hypothalamic disorders (51). Proposed mechanisms in this field are their involvement in both peripheral (vagal) and central (hindbrain and hypothalamus) pathways mediating satiety (52, 53) and also decrease in the food-induced reward signals and so decline of food-seeking behavior (53). In obesity and diabetes, glucose-dependent insulinotropic peptide (GIP)/GIPR and GLP-1 signaling are impaired. Restoring this damage results in a potential therapy for these diseases (52, 54, 55). Furthermore, their anti-obesity and glucose regulatory effects are linked to up-regulation of irisin, and so increase of muscle metabolism via AMP-activated protein kinase (AMPK) pathway (56). In the following some of the related studies are explained (Table 2).

*Preclinical studies*

Liraglutide possesses anti-atherogenic effects by suppressing macrophages foam cells formation related to blocking acyl-CoA: cholesterol acyltransferase 1 (57). 

Lixisenatide, the other GLP-1 analogue, by modulating STAT signaling pathway leads reprogramming of macrophages towards an M2 phenotype, decreases pro-inflammatory cytokine secretion and in turn reduces the atheroma plaque size and related cardiovascular events (58). 

Liraglutide attenuated a high-fat diet-induced atherosclerosis in apo E^-/-^ mice. In rat VSMCs culture, liraglutide inhibited angiotensin II-induced cell proliferation linked to activation of AMPK and in turn cell cycle arrest in G0/G1 phase (59). The blocking of GLP-1 signaling results in obesity in rats via decrease of nutrient-induced satiation (60). Over-expressing of GIP in mice improved insulin sensitivity, glucose tolerance, β cell function and reduced energy intake. Animals showed less weight gain and adiposity in response to high-fat diet (61).

According to a study on diet-induced obesity in mice, administration of exendin-4 resulted in improving effects attributed to the decrease in hepatic fibroblast growth factor 21 resistances. It is good to mention that hepatic fibroblast growth factor 21 evokes an important role in increasing insulin sensitivity, reducing triacylglycerol levels and hepatic steatosis, and improving glucose tolerance (62). Besides, exendin-4 evoked protective effects against maternal obesity-induced renal dysfunction in offsprings (63). 

Liraglutide administration to hyperlipidemic mice improved metabolic disorders, body weight as well as memory and learning (64). Besides, its chronic administration delayed the onset of diabetes and diabetes-related metabolic disorders including dyslipidemia, insulin resistance and weight gain (65). Liraglutide restored body weight as well as HOMA-IR (Insulin resistance index) via up-regulation of hepatic adenylate cyclase 3 level (66). It has been shown that liraglutide induces its beneficial effects on metabolism by driving white adipose tissue phenotype to brown phenotype via soluble guanylate cyclase- mediated pathway (67). 

Exenatide induces anti-inflammatory and anti-oxidant effects in high-fat-diet-induced obesity in rats. It evoked regulatory effects on fasting blood sugar (FBS), lipid profile, and induced weight loss. The beneficial effects of this drug were linked to up-regulation of hypothalamic insulin receptors. These receptors regulate appetite, white fat mass metabolism, and hepatic glucose output (68). 

*Clinical studies*

Prediabetes, T2DM, obese or overweighted individuals have lower GLP-1 responses to an oral glucose tolerance test (69). In a patient with Prader-Willi syndrome, who had been insufficiently controlled with insulin therapy, adding exenatide improved glycaemia, reduced weight gain and restored blood pressure, microalbuminuria, glycosylated hemoglobin and lipid profile (70). Besides, exenatide efficacy on lipid profile (62) and extreme obesity in pediatrics (71) has been proven in another studies. 

In comparison with increasing insulin doses, adding liraglutide to obese T2DM patients not only had a good glycemic control but also possessed good effects on the control of body weight gain (72). 

GLP-1 agonists, exenatide or liraglutide, could be engaged as an important modulatory therapy in patients with morbid hypothalamic obesity by controlling appetite (73-75). Folli and Mendoza (2011) have reviewed the potential effects of exenatide for the treatment of obesity. According to the literate data, in obese patients with/without T2DM, administration of exenatide decreased body weight, improved glycemic control and reduced blood pressure and body fat mass (76). It has promising effects in T2DM patients who suffered from weight gain and had insufficiently glycemic control on insulin therapy (77). Exenatide has been proposed as a good treatment in the reduction of liver and epicardial fat content in obese patients with T2DM (78). The beneficial effects of exenatide treatment on the decrease of body weight and restoration of FBS and HbA1c in T2DM patient were linked to up-regulation of irisin (56) and delay in the gastric emptying time (79). It had been discussed that irisin has an fundamental role in glucose metabolism by stimulating membrane translocation of glucose transporter type 4 and AMPK phosphorylation (80). Besides, exenatide treatment reduces bone morphogenetic protein-4 level that is a regulator of white adipogenesis, independently to weight loss (81). 

In comparison with orlistat, liraglutide is a valuable option to improve the success of weight loss in obese and overweight individuals without diabetes (82). Administration of liraglutide to obese and non-diabetic patients with sleep apnea, decreased apnea in relation to reduction of body weight (83). Besides, it showed a good efficacy in reducing body mass index and fat mass with increase in skeletal muscle index in overweight and obese T2DM patients (84) and improvement of postprandial lipaemia in T2DM patients independently to gastric emptying (85). Not only in adult patients, but also in adolescents, liraglutide administration had good impacts in controlling obesity with similar safety and tolerability (86). 

Administration of GLP-1 agonists not only in T2DM but also in type 1 diabetes mellitus, evokes worth effects in glycemic control and decreasing body weight (87).

According to the clinical data, the combination therapy of metformin plus exenatide is a worth therapy for reducing intra-abdominal fat content and inflammatory states and restoring insulin resistance in obese patients with T2DM (88). Surprisingly, this combination therapy is more effective in overweight and obese women than in men patients (89). 

Furthermore, a meta-analysis of randomized controlled trials study have proved the efficacy of GLP-1RAs in decreasing C-reactive protein, an inflammatory marker, in patients with T2DM (90). 

*Effects on the high blood pressure*

GLP-1RAs that are used to treat T2DM have beneficial effects on the cardiovascular system in chronic exposure. Minor rise in blood pressure and heart rate that is seen in short-term exposure with these agents is linked to CNS pathways. However, after intermediate- or long-term exposure these effects are compensated. Totally, anti-hypertensive effects of GLP-1RAs is classified into renal- and non-renal-mediated mechanisms. GLP-1RAs increase atrial natriuretic peptide (ANP) level by effects on atrial cardiomyocytes that in turn evokes cyclic guanosine monophosphate and NO production and hence vascular relaxation. Besides, ANP elevation promotes sodium excretion and natriuresis. A decrease in the plasma renin activity is the other proposed mechanisms. Furthermore, the decrease in blood presser with this class is considered as an outcome of weight loss, inhibition of intestinal salt absorption, central inhibition of salt intake, endothelial-dependent vasodilation and direct action on vascular smooth muscles (91, 92). In the following some of the related studies are explained (Table 3).

*Preclinical studies*

GLP-1RAs are associated with a modest reduction in blood pressure and a slight increase in heart rate, but no significant association with hypertension (93). AC3174, the exenatide analogue, better than GLP-1 evoked anti-hypertensive, cardio protective and renoprotective as well as insulin-sensitizing effects in Dahl salt-sensitive rats. It has been proposed as a good choice for increasing survival in cardiorenal syndrome and hypertension (94). Liraglutide administration is a valuable engaged therapy in T2DM, hypertension associated with polycystic ovary syndrome (95) and pulmonary hypertension (96).

*In vitro* and mice studies revealed that the hypotensive effects of liraglutide are a GLP-1 and ANP mediated mechanisms. It has been verified that gut GLP-1 by influence on atrial GLP-1 receptors directly enhances cardiac ANP secretion and in turn relaxes vascular tone and indirectly increases sodium/creatinine urine exertion and so evokes hypotension (97).

*Clinical studies*

Some clinical studies proposed the modulatory effects of exenatide on systolic blood pressure, especially in patients with T2DM and hypertension (98, 99). However, a single dose administration of PF-04603629, a long-acting GLP-1 mimetic compound, increased pulse rate and mean diastolic blood pressure that not exceed from normal range (100). 

One clinical study on healthy volunteers verified that intestinal glucose load possesses hypotensive potential effects by influence on GLP-1 release (101). In comparison with glimepiride, treatment with liraglutide is superior especially in T2DM patients with weight gain, hypoglycemic episodes, and hypertension (102). 

Based on a clinical study, the blood pressure-lowering effects of exenatide have been attributed to its effects on glycaemia and body weight (103), where the later study excluded its relation with weight loss or improvement in HbA1c (104). 

Systematic and meta-analysis verified the blood pressure (104-107) and weight (104, 106) lowering effects of liraglutide and exenatide in T2DM patients. Besides, it has been confirmed that GLP-1 consumption is associated with the rise in heart rate (104, 106) and in compare with exenatide, heart rate rises were greater with liraglutide (106). The safety and efficacy of liraglutide in T2DM patients who are on peritoneal dialysis have been proved in controlling glucose levels, glycemic fluctuations, blood pressure and improving left ventricular functions (108). The efficacy of 36-month liraglutide therapy has been evaluated in a retrospective study on T2DM patients. Based on the gathered data, liraglutide had preserved its effects on controlling body weight, metabolic factors, LDL, blood pressure and waist circumference after 3 years. Besides, it had no major effects on heart rate and renal parameters (109). Furthermore, in chronic heart failure patients without T2DM, administration of liraglutide reduced body weight, HbA1c and 2-hr glucose tolerance test with no effects on myocardial glucose uptake, myocardial blood flow, and myocardial blood flow reserve (110). The other clinical study on T2DM patients with stable coronary artery disease monitored the effects of liraglutide on 24 hr ambulatory blood pressure and showed it has no blood pressure lowering effects but enhances 24 hr heart rate (111).

Administration of dulaglutide to hypertensive T2DM patients is accompanied by the decrease of systolic blood pressure and pulse pressure and rise of heart rate (112). According to a clinical data on well-controlled T2DM patients, the intravenous administration of exenatide is accompanied by the increase of heart rate and mean atrial pressure during intraduodenal infusion of glucose (113). 

*Effects on the serum glucose level*

GLP-1RAs are the newest class of anti-diabetic agents. So many studies have proved their effectiveness in this field. Herein, we mainly notice mechanistically to their superior effects in glycemic control. Both GLP-1 analogues and DPP-4 inhibitors reduce HbA1c. However, GLP-1 analogues are more effective in weight loss and glycemic lowering (114). Administration of GLP-1 analogues with basal insulin addresses beneficial effects in T2DM patients who are overweight and uncontrolled on oral anti-diabetic drugs or basal insulin (115). GLP-1RAs promote autophagy by modulating AMPK activity that leads to reserve pancreas β-cell mass and its protection against glucotoxicity (116). It has been proved that delayed timing of post-challenge peak glucose relates with deteriorating pancreas β-cell function and worsening oral glucose tolerance test. Liraglutide by increasing pancreas β-cell function improves oral glucose tolerance test and shifts the timing of peak serum glucose earlier (117). 

Administration of liraglutide (1.2 mg/day for 6 months) decreases body weight, HbA1c and liver fat content (118). 

In comparison with patients treated with basal inulin, exenatide administration results in similar glycemic control with greater weight reduction (119). GLP-1 agonists cause in a good control on postprandial glucose level. Exenatide administration to metformin-treated T2DM patients improved daily glucose control, FBS and postprandial glucose level (120). This drug by decreasing the gastric emptying rate has an important role in postprandial plasma glucose level (121). 

Semaglutide significantly restores lipid metabolism, fasting, and postprandial glucose level. Although the gastric emptying rate is totally similar between semaglutide and placebo, the first-hr delay with this drug may decrease the rate of glucose entry into the circulation (122). Furthermore, the addition of lixisenatide, a short-acting GLP-1 agonist, to insulin-treated T2DM patients leads a good control on HbA1c levels by slowing gastric emptying and reducing postprandial glucose excursions (123). 

In insulin-treated T2DM patients on hemodialysis, dulaglutide improved glycemic control and evoked a decrease in insulin daily dose (124). Administration of dulaglutide was accompanied by the reduction of body weight, especially in women. Although it restored HbA1c and FBS level without regard to gender, greater reduction in HbA1c and FBS level and modest hypoglycemia incidence had been seen in patients with a higher HbA1c baseline (125). It is important to consider that chronic administration of liraglutide to healthy volunteer does not result in tolerance to its glucose-lowering effects (126). 

**Figure 1:**
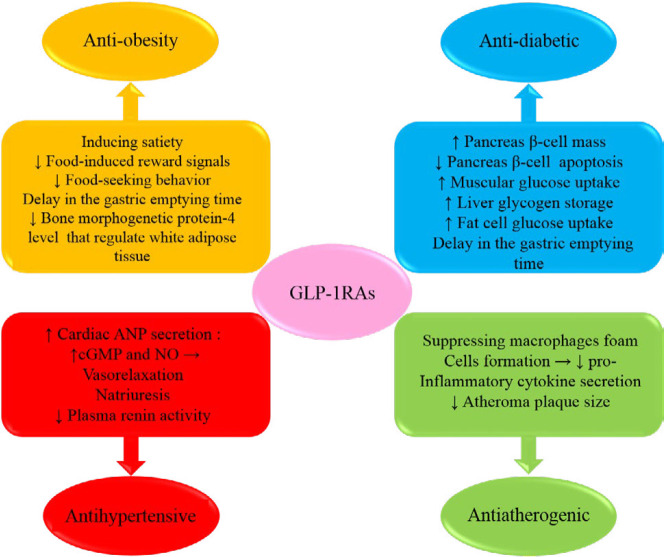
Schematic description showing the mechanisms of glucagon-like peptide-1 receptor agonists in the treatment of some metabolic syndrome components (obesity, hypertension and diabetes) and outcomes (atherosclerosis)

GLP-1RAs: glucagon-like peptide-1 receptor agonists, ANP: atrial natriuretic peptide, cGMP: cyclic guanosine monophosphate, NO: nitric oxide

**Table 1:** Comparing glucagon-like peptide-1 receptor agonists based on efficacy in glycemic control and gastrointestinal side effects

Efficacy in glycemic control	Semaglutide (H, L) > Dulaglutide (H, L) = Liraglutide (H, S) > long-acting Exenatide (E, L), Albiglutide (H, L) > twice a day Exenatide (E, S) = Lixisenatide (E, S)
GI side effects	twice a day Exenatide (E, S) > Lixisenatide^1^ (E, S), long-acting Exenatide (E, L) > Liraglutide (H, S), Dulaglutide (H, L) > Albiglutide (H, L)

H: a derivative of human glucagon-like peptide-1 (GLP-1); E: a derivative of exendin-4; L: long-acting GLP-1 receptor agonist with several days half life; S: short-acting GLP-1 receptor agonist with 2-12 hr half life; GI: gastrointestinal

^1^ just in compare with exenatide twice daily but not liraglutide

**Table 2:** The effects of glucagon-like peptide-1 receptor agonists on lipid profile, body weight and related complications

Study design		Maine results	Ref.
*In vitro* studies	Primary cultured human monocyte-derived macrophages GLP-1^1^ (0.5-10 nM), GIP^2^ (0.5-10 nM), exendin (10-150 nM) or liraglutide (10-150 nM)	in some concentrations: ↓ cholesterol ester accumulation ↓ ACAT1^3^ expression ↑ ABCA1 expression ↓ CD36 expression (but not with liraglutide)	(57)
	Murine bone-marrow-derived macrophages lixisenatide 40 nM for 7 days, LPS 100 ng/ml for the last 24 h or 6h.	↓ IL-6^4^ secretion in the supernatant stimulation with LPS for 6 h: ↓ pSTAT1/STAT1^5^ ↑ pSTAT3/STAT3 ↓ iNOS^6^/α-tubolin (M1 macrophage marker) stimulated with LPS^7^ for 24 h: ↑ arginase I/α-tubolin (M2 macrophage marker)	(58)
	Pre adipocyte 3T3-Li cells Lirglutide (10, 100 µM) for 3 days	↓ size of droplet after 10 days ↑ all brown fat markers examined, including Cidea, PPARγ^8^, PRDM16^9^ and UCP-1^10^, as well as mitochondrial markers (CytoC^11^, PGC1α^12^ and TFAM^13^) and COX-IV^14^	(67)
Animal studies	Male apo E^-/-^ mice on high-fat diet Liraglutide 107 nmol/kg/day, for 4 weeks	↓ body weight ↑ GLP-1 ↓ surface area of atherosclerosis lesions in aorta ↓ monocyte/macrophage accumulation in aorta	(57)
	Apo E^-/-^ Irs2^+/- 15^ on atherogenic diet for 2 months Lixisenatide, 10 µg/kg/day or liraglutide 400 µg/kg/day, during the last month of diet	↓ blood pressure ↓ fasting plasma insulin improved glucose metabolism ↓ atherosclerotic lesions in aortic arch ↓ macrophages, T-lymphocytes, collagen area and, necrotic core area in atheroma ↑ fibrosis cap thickness in the atheroma area ↓ circulating plasma level of IL-6 ↑ arginase I in plaques (M2 macrophage marker) ↓ iNOS in plaques (M1 macrophages marker) ↑ arginase I-/iNOS-stained area in atheroma	(58)
	Apo E^-/-^ mice on high-fat diet Liraglutide, 400 mcg/day for 4 weeks	in the aortic wall: ↓ atherosclerotic lesions ↑ AMPK^16^ activation	(59)
		in isolated aorta: ↑ relaxation responses to acetylcholine	
	Female Sprague-Dawley rats on high-fat diet for 6 weeks, prior to pregnancy, during pregnancy and lactation, then their offspring were weaned to high-fat diet Exendin	in the kidneys: ↓ inflammation ↓ oxidative stress ↓ fibrosis	(63)
	Male Swiss mice on high-fat diet for 20 weeks Liraglutide, 200 µg/kg, BID^17^, for 28 days	↓ body weight ↓ energy intake improving non fasting glucose normalizing glucose tolerance test ↑ recognition index ↑ learning and memory ability	(64)
	UC Davis Type 2 Diabetes Mellitus (UCD-T2DM) rat Liraglutide 0.2 mg/kg, BID, 4 months	the onset of diabetes was delayed ↓ diabetes incidence ↓ non-fasting blood glucose ↓ FBS^18^ ↓ HbA1c^19^ ↓ body fat ↓ lean body mass ↓ body ash ↓ plasma insulin ↓ body weight ↓ energy intake ↓ glycosuria ↓ albuminuria ↓ 24-h urine volume ↓ triglyceride ↑ adiponectin	(65)
	High-fat diet induced diabetes and obesity in db/db and C57BL/6j mice, respectively Liraglutide 0.2 mg/kg/ day, for 12 weeks	↓ body weight ↓ HOMA-IR^20^ ↑ hepatic adenylate cyclase 3	(66)
	High-fat diet induced obesity in KKAy mice Liraglutide 400 µg/kg/day, for 12 weeks	↓ body weight ↓ adipocyte size in white fat ↑ the white fat browning ↑ mitochondrial biogenesis in white fat ↑ soluble guanylate cyclase-dependent pathway	(67)
	High-fat diet induced obesity in rats Exenatide, 10 µg/kg, for one month	↓ body weight ↓ FBS ↓ insulin level ↓ insulin resistance ↓ dyslipidemia ↓ oxidative stress ↓ TNF-α^21^ serum level ↑ hypothalamic insulin receptor	(68)
Clinical study	T2DM^22^ on a weight-maintaining diet, 3 weeks before the study Exenatide 5-10 μg, BID and pioglitazone 30-45 mg/day orally for 52 weeks	↓ hepatic fat content ↓ plasma FGF21^23^ concentration	(62)
	Children and adolescents (age 9-16 years old) with extreme obesity Exenatide 5-10 µg, BID, for 3 months	↓ BMI^24^ ↓ body weight ↓ fasting insulin improved OGTT^25^ and β ano function	(71)
	Obese T2DM patients Liraglutide 0.6-1.2 mg/day, for 12 weeks	good glycemic control ↓ daily insulin dose ↓ hypoglycemic events ↓ body weight and waist circumference	(72)
	Patients with hypothalamic obesity Exenatide 10 µg, BID, for 52 weeks	↓ body weight but not significantly	(75)
	T2DM patients with obesity, progressive weight gain, and insufficiently glycemic control on insulin therapy Exenatide 5 µg, BID, for 12 months	↓ body weight ↓ insulin doses	(77)
	T2DM uncontrolled on oral antidiabetic drugs Exenatide, for 26 weeks	↓ HbA1c ↓ weight ↓ epicardial adipose tissue ↓ hepatic triglyceride content	(78)
	T2DM with obesity Exenatide, for 12 weeks	↑ irisin ↓ FBG ↓ HbA1c ↓ BMI	(56)
	Adults 18 years or older, obesity class I and II Exenatide 5 µg, BID, for 30 days	delayed the gastric emptying of solids ↓ caloric intake ↑ weight loss (not significant)	(79)
	Non-diabetic participants with obesity with moderate to severe obstructive sleep apnea Liraglutide 3.0 mg, 32 weeks both as adjunct to diet (500 kcal^26^ day -1 deficit) and exercise	↓ apnea-hypopnea index ↓ weight ↓ HbA 1c ↓ systolic blood pressure	(83)
	Overweight and obese elderly with T2DM Liraglutide up to 3 mg/day for 24 weeks	↓ body mass index ↓ weight ↓ fat mass ↓ HbA1c ↑ glycemic control ↑ skeletal muscle index	(84)
	T2DM patients Liraglutide 1.8 mg/day, for 21 days	↓ postprandial triglyceride, apoB48 and glucose ↑ postprandial insulin and C-peptide ↓ FBS ↑ fasting insulin ↑ fasting C-peptide	(85)
	T1DM on insulin and sub-optimal glycaemic control or obesity A GLP-1 analogue was added to pre-existing treatment	↓HbA_1c_ ↓weight	(87)

^1^ Glucagon like peptide; ^2^ Gastric inhibitory polypeptide; ^3^ Acetyl-CoA acetyltransferase; ^4^ Interleukin 6; ^5^ Signal transducer and activator of transcription; ^6^ Inducible nitric oxide synthase; ^7^ Lipopolysaccharide; ^8^ Peroxisome proliferator-activated receptor gamma; ^9^ PR domain containing 16; ^10^ Mitochondrial uncoupling proteins; ^11 ^cytc; ^12^ Peroxisome proliferator-activated receptor-gamma coactivator; ^13^ Mitochondrial transcription factor A; ^14^ Cytochrome c oxidase subunit 4; ^15^ Irs2; ^16^ 5’ adenosine monophosphate-activated protein kinase; ^17^ BID; ^18^ Fast blood sugar; ^19^ Hemoglobin A1c; ^20^ Homeostatic model assessment for insulin resistance; ^21^ Tumor necrosis factor-α; ^22^ Type 2 diabetes mellitus; ^23^ Fibroblast growth factor 21; ^24^ body mass index; ^25^ oral glucose tolerance test**; **^26^ kilocalorie

**Table 3:** The effects of glucagon-like peptide-1 receptor agonists on the high blood pressure

Study design			results	refer
Animal study	Dahl salt-sensitive rats were fed with high-salt chow and AC3174, SC^1^ infusion, or GLP-1^2^, 4 weeks		↓ SBP^3^ ↓ left ventricular wall stress ↓ left ventricular mass and serum creatinine (just with AC3174) ↓ fasting insulin ↓ HOMA^4^ index ↑ creatinine clearance rate improvement of high salt diet-renal sclerosis	(94)
	Pre-pubertal female Sprague Dawley rats between 4-5 weeks of age implanted S.C. with DHT^5^ pellets (90 day release; 83μg/day), after 12 weeks of age, received liraglutide 0.2 mg/kg, S.C., BID^6^ for 4 weeks		↓ body weight improved glucose tolerance test improved mean atrial pressure	(95)
	GLP-1r^-/-^ mice	Ang II^7^, S.C., for 3 weeks followed by liraglutide	no effects on systolic or diastolic blood pressure no effects on the plasma ANP^8^ concentration	(97)
		isolated mice heart	liraglutide has no effects on concentration of ANP in heart perfusate	
	NPPa^-/-^ mice (ANP knockout)	Ang II, S.C., for 3 weeks followed by liraglutide	liraglutide has no effects on blood pressure and sodium/creatinine in urine	
	wild type mice	Ang II, for 3 weeks followed by liraglutide,	↑ plasma ANP concentration	
			↓ blood pressure ↑ sodium/creatinine in urine	
		Exendin_9–39_ (a GLP-1R antagonist), or L-NMMA^9^ or anantin^10^ for 2 days, followed by liraglutide	both exendin_9–39_ and anantin blocked the antihypertensive actions of liraglutide, whereas l-NMMA had no effect	
		in isolated aorta pre-contracted with phenylephrine	Ach^11^: produced dose dependent relaxation, ↑ p-eNOS, vasodilator stimulated phosphoprotein and cGMP^12^ in aorta liraglutide had none of aforementioned effects	
Clinical studies	T2DM^18^, Exenatide, 5 µg for 4 weeks followed by 10 µg, BID for 12 weeks, S.C.		liraglutide increased concentration of ANP in perfusate a slight but not significant decreasing effects on SBP	(98)
	T2DM PF-04603629, 1, 3, 10, 20, 40, 50 and 70 mg, single dose, S.C.		after 24 h a dose related but not significant increasing effects on pulse rate and DBP^13^	(100)
	T2DM Liraglutide 0.6, 1.2 or 1.8 mg single dose with metformin		↓ HbA1c^14^ ↓ FBG^15^ ↓ PPG^16^ ↓ body weight ↓ hypoglycemia episodes ↓ SBP	(102)
	T2DM and peritoneal dialysis Liraglutide for 12 months		↓ HbA1c ↓ glycosylated albumin ↓ fasting/postprandial glucose level ↓ daily glucose level ↓ glycemic fluctuation ↓ SBP ↓ left ventricular mass index ↑ left ventricular ejection pressure	(108)
	T2DM and HTN^17^ Dulaglutide, 1.5 or 0.75 mg, single a week, for 26 week, S.C.		↓ diurnal and nocturnal SBP and pulse pressure ↑ diurnal and nocturnal heart rate	(112).

^1^ Subcutaneous injection; ^2^ Glucagon-like peptide-1; ^3^ Systolic blood pressure; ^4^ Homeostatic model assessment; ^5^ Dihydrotestosterone; ^6^ Two times a day; ^7^ Angiotensin II; ^8^ Atrial natriuretic peptide; ^9^ A nitric oxide synthase inhibitor; ^10^ A natriuretic peptide receptor antagonist); ^11^ Acetylcholine; ^12^ Cyclic guanosine monophosphate; ^13^ Diastolic blood pressure; ^14^ Hemoglobin A1c; ^15^ Fast blood glucose; ^16^ Post prandial glucose; ^17^ Hypertension; ^18^ Type 2 diabetes mellitus

## Discussion

Nowadays, metabolic syndrome and its associated complications are considered as the most important health problem worldwide. It is estimated to affect over a billion people in the world (127), hence it imposes great healthcare burden globally. Lifestyle modification and caloric restriction remain the primary tools for managing its predisposing disorders including dyslipidemia and insulin resistance(13, 14). However, in severe conditions, pharmacological interventions may be required to control lipid abnormalities, hypertension and glucose intolerances in which a large number of pharmacological options are present. In this regard, DPP-4 inhibitors, sodium-glucose cotransporter 2 inhibitors, and GLP-1 receptor agonists are proposed for glucose-lowering medications. Orlistat, phentermine/topiramate, lorcaserin, naltrexone sustained release/bupropion sustained release and liraglutide are suggested for body weight reduction. This kind of combination therapy imposes great health-cost, increases the possibility of drug interactions and related side effects, and therefore decreases patients’ compliance (9). Introducing pleiotropic drugs that control all aspects of metabolic syndrome as a single disease is a key area for improvement in managing MetS and increasing patients’ compliance. To the best of our knowledge, there is a gap in introducing and approving polypharmacological drugs that manage MetS as a whole.

Drug discovery in traditional drug development usually takes 10-15 years and the success rate is very low. Furthermore, it needs high investments and is very expensive. Drug repositioning that becomes popular in recent years involves investigating and approving new therapeutic uses for old drugs. In comparison with drug discovery, drug repositioning is an efficient, economical and riskless process (128).

The drug repositioning strategy includes three steps. First, the identification of an appropriate drug for the diseases; then, mechanistic evaluation of its effect in preclinical studies; and finally, efficacy assessment in phase II clinical trials. Of these three steps, step 1 “finding novel drug-disease relationships” is the gold matter in the drug repositioning (129).

GLP-1RAs are one of the newest pharmacological interventions in managing diabetes with great glycemic control especially in overweight T2DM patients who are uncontrolled with the other anti-diabetic drugs. According to both preclinical (*in vitro* and animal) and clinical studies, GLP-1RAs by effects on peripheral and central pathways induce satiety, decrease body weight and control dyslipidemia. They induce p-AMPK activation, decrease pro-inflammatory conditions and evoke anti-atherogenic effects. Chronic administration of GLP-1RAs overcome hypertension by renal- and non-renal mediated mechanisms. (Figure 1). However, these effects are modest and limited to some levels. 

Herein, we proved our hypothesis “the effectiveness of GLP-1RAs in MetS”, showed this relation and solved the first step of drug repositioning. In the future, Long-term randomized clinical trial results are needed to validate these preliminary data, and post-marketing evaluation is necessary to verify their safety, especially during pregnancy, breastfeeding, and susceptible people. Furthermore, reformulation and synthetization newly design chemicals of GLP-1RAs and/or combination with other drug/s might improve their efficacy on MetS components. Approving and adding this kind of drugs as an adjuvant or main therapy to therapeutic guidelines decrease side effects and the risk of drug interactions, and increase patients’ compliances. This category of pharmacological drugs, GLP-1RAs, may reach researchers of MetS to their ultimate goal: managing MetS as a single disease. It seems hard but not impossible to develop new drugs with polypharmacological effects on MetS component as a single condition in the future. 
